# Vascular geometry as a risk factor for non-penetrating traumatic injuries of the aortic arch

**DOI:** 10.1371/journal.pone.0180066

**Published:** 2017-06-23

**Authors:** Andreas Schicho, Lukas Luerken, Christian Stroszczynski, Ramona Meier, Andreas G. Schreyer, Lena-Marie Dendl, Stephan Schleder

**Affiliations:** Department of Radiology, University Medical Center Regensburg, Regensburg, Germany; University of Florida, UNITED STATES

## Abstract

**Purpose:**

To assess biomechanical factors in aortic arch geometry contributing to the development of non-penetrating aortic arch injury (NAAI) in multiply injured patients with an Injury Severity Score (ISS) ≥ 16.

**Material and methods:**

230 consecutive multiply injured trauma patients with an ISS ≥ 16 admitted to our Level-I trauma center during a consecutive 24-month period were prospectively included of whom 13 presented with NAAI (5.7%). Standardized whole-body CT in a 2x128-detector-row scanner included a head-and-neck CTA. Aortic arch diameters, width, height, angles and thoracic width and height were measured in individuals with NAAI and ISS-, sex-, age-, and trauma mechanism-matched controls.

**Results:**

There was no difference between groups regarding sex, age, ISS, and aortic diameters. The aortic arch angle in individuals with NAAI (71.3° ± 14.9°) was larger than in healthy control (60.7° ± 8.6°; p*<0.05). In patients with NAAI, the distance between ascendent and descendent aorta was larger (5.2 cm ± 1.9 cm) than in control (2.8 ± 0.5 cm; ***p<0.001). The aortic arch is higher above tracheal bifurcation in NAAI (3.6 cm ± 0.6 cm) than in matched control (2.4 cm ± 0.3 cm; ***p<0.001). Accordingly, the area under the aortic arch, calculated as half of an eliptic shape, is significantly larger in patients with NAAI (15.0 cm2 ± 6.5 cm2) when compared to age- and sex-matched controls without NAAI (5.5 cm2 ± 1.3 cm2; ***p<0.001).

**Conclusion:**

Besides the magnitude of deceleration and direction of impact, width and height of the aortic arch are the 3rd and 4th factor directly contributing to the risk of developing traumatic NAAI in severely injured patients.

## Introduction

Traumatic, non-penetrating aortic arch injuries (NAAI) are frequently fatal; despite advances in prehospital emergency services, up to 80% of patients with NAAI do not reach the hospital [1,2]. A sudden deceleration of the thorax, eventually followed by a re-acceleration („whiplash“), is believed to be the underlying mechanism therein [1]. Factors known to contribute to the development of NAAI is the magnitude of deceleration [3] and direction of impact, with lateral impacts being more likely to develop NAAI [4]. Deceleration is the loss of speed (v) in a distinct time (t; Δv/Δt). With respect to basic principles of physics, such as Newton’s 2^nd^ law of motion, moment of inertia, and law of Laplace, further factors should contribute to the risk of development of NAAI; aortic arch geometry, blood pressure, and direction of force can add up (theory of superposition) and cause a tear in the aortic wall. While blood pressure, heart cycle and resulting flow patterns [5] can’t be determined retrospectively, the aortic arch geometry is well documented in standardized computed tomography (CT). Purpose of our study was to reveal distinct geometric properties of the aortic arch in patients with NAAI in comparison to age- and sex-matched healthy controls to gain further insights to the biomechanics in the development of traumatic NAAI.

## Material and methods

230 consecutive multiply injured trauma patients admitted to our Level-I trauma center during a consecutive 24-month period were prospectively included. Patients of the control group were matched by ISS, age, sex, and trauma mechanism (high deceleration/acceleration trauma) from the same cohort[6]. The study was approved by the institutional review board. All patients underwent a standardized whole-body CT scan in a 2x128-detector-row scanner (Siemens SOMATOM Definition Flash, Siemens Healthcare AG, Forchheim, Germany), consisting of a non-contrast head CT and a one-phase contrast-enhanced whole body CT.

The Independent Ethics Committee at the Regensburg University confirmed, that for the scientific project no ethics-approval or commission’s opinion was necessary due to the fact, that according to our applicable laws and guidelines such anonymized retrospective study without any study-related clinical intervention or use of patients’ personal data does not have to be submitted to the ethics committee. Thus, no informed consent was obtained.

### CT scan

Initially, a non contrast-enhanced scan of the neurocranium was acquired in .75 mm slices (360 mAs, 120 kV, pitch .55, increment .75 mm, FoV 230 mm).

Aiming at a scanning time as short as reasonable possible in the setting of a routine integration of CT into emergency room algorithms, we use a single contrast-enhanced scan from head-to-toe with 120 ml of contrast agent (Accupaque 350; GE Healthcare Buchler GmbH & Co. KG, Braunschweig, Germany) injected automatically followed by a flush with 30 ml NaCl, both at injection rates of 3 ml/s. The scan is started at a fixed delay of 55 seconds after contrast-agent injection is completed. The FoV is 500 mm, slice thickness 5.0 mm, increment 5.0 mm, pitch .6, kV and mAs are calculated and set automatically (CARE kV and CARE Dose; Siemens Healthcare AG, Forchheim, Germany) based on the tomogram. A soft-tissue kernel with medium edge attenuation (B26f medium ASA) was used for calculation of axial, coronal, and sagittal views of the head and neck. Additional reconstruction were rendered using a B60f kernel for lung tissue (axial), B60f sharp kernel for bones (axial and coronal, additional sagittal for spine), and B30f for soft tissue (axial, additional coronal for abdomen). Further reconstruction were calculated on the radiologists discretion depending on the findings or suspicions drawn from the standard data sets.

Image interpretation was performed using a standard three-monitor PACS work-station using Syngo and SyngoVia (Siemens Healthcare AG, Forchheim, Germany).

### Aortic arch geometry measurements and calculations

First, the horizontal plane was set at the first slice showing the separation of left- and right-main bronchus (tracheal bifurcation). Second, the sagittal plane was set to pass the center of ascendent and descendent aorta on the horizontal plane. Third, the sagittal plane was tilted sideways until it depicted the origin of the left subclavian artery with its maximum sagittal diameter.

**Fig 1:**
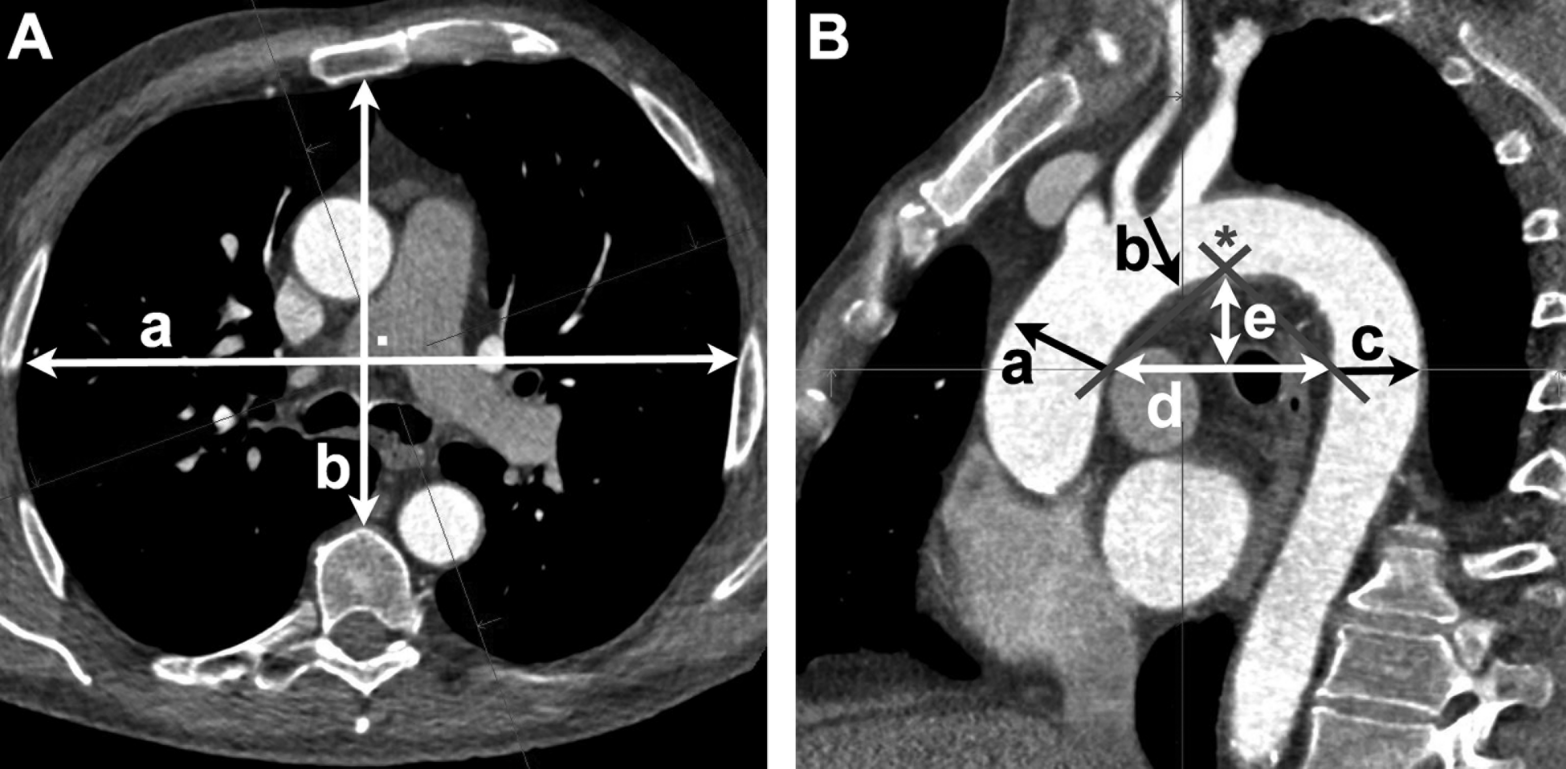
Example of measures. A + B are correspondent planes set with SyngoVia imaging software (Siemens Healthcare AG, Forchheim, Germany). A) Maximum width (a) and depth (b; perpendicular to a) of thorax on the plane of tracheal bifurcation. B) Diameters of Aorta ascendens (a), aortic arch (b), and Aorta descendens (c). Aortic arch index (AAI) is the distance (d) of the inner walls of the ascendent and descendent aorta. The height (e) was measured perpendicular to (d) at the highest point of arch closest to the mid of (d). At the top of the delineated triangle, the aortic arch angle (AAA; *) was measured.

Aortic arch index (AAI) was measured as the shortest distance between ascendent and descendent aorta at the level of the tracheal bifurcation (Fig 1). Aortic arch angle (AAA) was measured from the inner wall of the aorta at its highest point in the arch to the line drawn for the AAI, as comparably described previously [7]. Diameters of Aorta ascendens and descendens were measured perpendicular to the inner wall starting at tracheal bifurcation. Diameter of aortic arch was measured perpendicular from the outlet of the left subclavian artery. Height of the aortic arch (AAH) was measured perpendicular from the AAI to the highest point of the aortic arch at its inner wall.

Depth and width of the thorax were also measured on level of the tracheal bifurcation. Thorax transection area was calculated as (1/2 x width) x (1/2 x depth) x π, assuming an eliptic shape. Calculation of the area under the arch was as follows: ((1/2 x AAI) x (AAH) x π) x 0.5.

### Statistical analysis

For statistical calculations, analysis, and plotting, GraphPad Prism version 5.00a for Mac (GraphPad Software, San Diego California, U.S.A.) was used. Groups were compared using two-sided student’s t-test. Statistical significant differences were regarded at p <0.05.

## Results

Of 230 patients with ISS ≥ 16, we found 13 individuals with NAAI (5.7%). For baseline characteristics of patients with NAAI and their ISS-, age-, sex-, and trauma mechanism-matched controls, taken from the group of 217 without NAAI, refer to Table 1. There were no statistical significant differences regarding age, sex, and ISS. Injury mechanism in all patients (NAAI and control) included a sudden deceleration/acceleration (car accidents, motorcycle accidents, falls from height >3m, hit by car as pedestrian).

**Table 1:** Baseline characteristics of individuals with NAAI and without (control).

	control	NAAI	*p*
n	13	13	*n*.*s*.
Age (mean ± SD; min; max)	44.3 ± 15.0; 19; 74	44.4 ± 14.8; 19; 73	*n*.*s*.
Sex (m/f)	11/2	11/2	*n*.*s*.
ISS (mean ± SD)	33 ± 12.9	38 ± 12.4	*n*.*s*.

### Aortic arch diameters and thorax transection area

**Fig 2:**
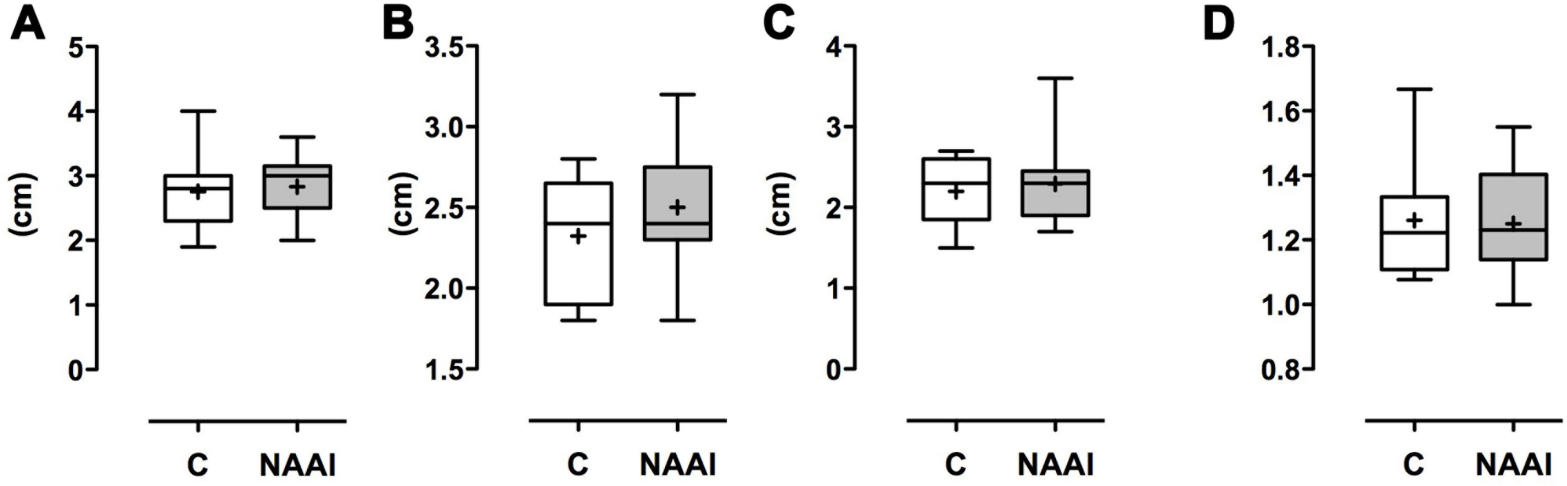
Diameters of the aortic arch. A) Diameter of Aorta ascendens, Aortic arch (B), and Aorta descendens (C). While diameters in NAAI tend to be larger than in control, the ratio of Aorta ascendens/Aorta descendens is quite similar in both groups (D).

**Fig 3:**
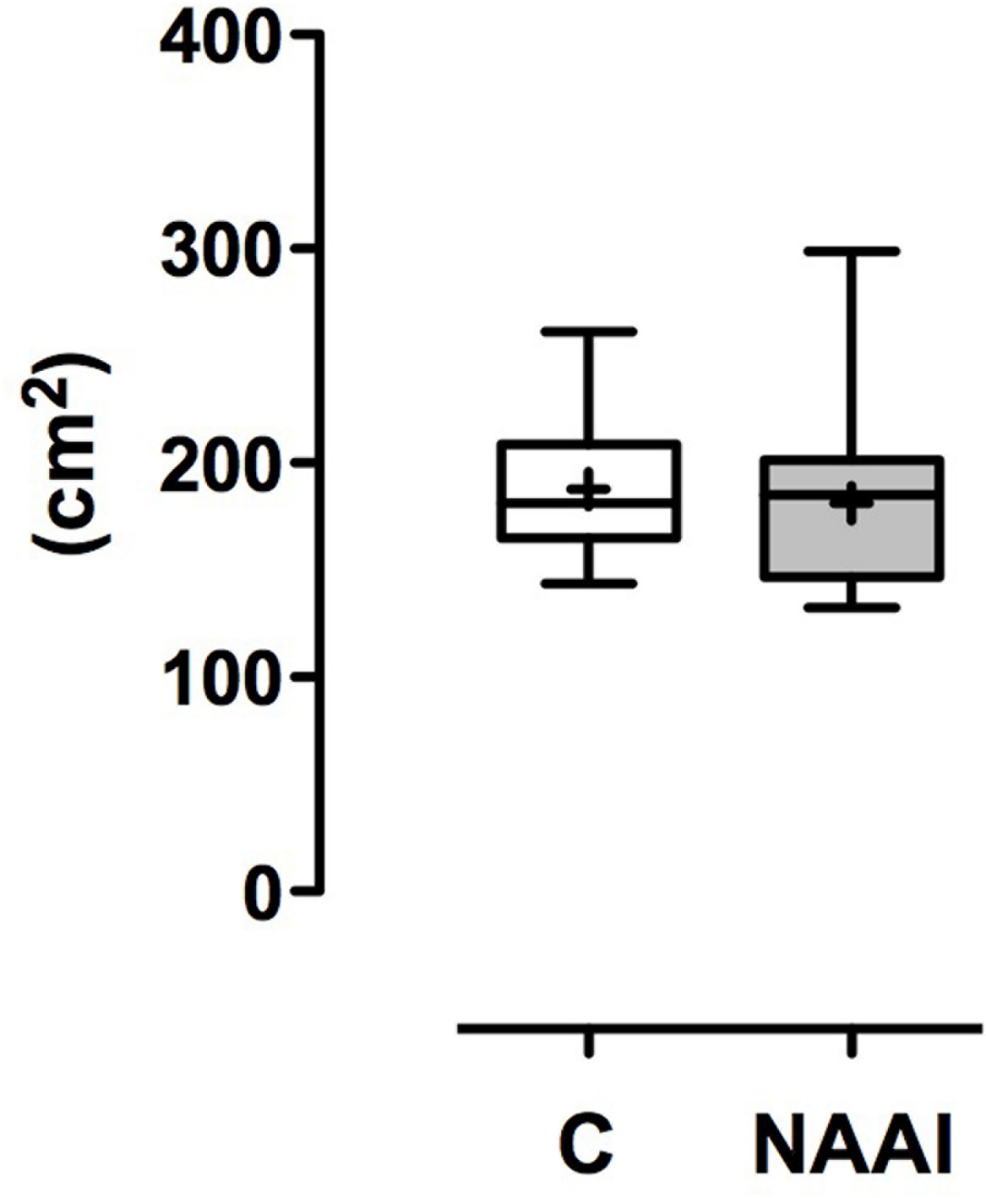
Area of transverse thorax transection.

Average diameters for the A. ascendens, aortic arch, and A. descendens showed no statistically significant difference (Table 2; Fig 2); the ratio of A. ascendens and A. descendens diameters was even more similar in both groups (control: 1.26 ± 0.18 vs. NAAI: 1.25 ± 0.17). The area of transverse thorax transection calculated as eliptic shape is not of statistical difference, either (Table 3; Fig 3).

**Table 2:** Diameters of A. ascendens, A. arch, and A. descendens and ratio of A. asc. / A. desc. Mean ± SD. n.s. = no significant difference.

	control	NAAI	*p*
Diameter A. asc. (cm)	2.8 ± 0.6	2.8 ± 0.5	*n*.*s*.
Diameter A. arch (cm)	2.3 ± 0.4	2.5 ± 0.4	*n*.*s*.
Diameter A. desc. (cm)	2.2 ± 0.4	2,3 ± 0.5	*n*.*s*.
Ratio A. asc. / A. desc.	1.26 ± 0.18	1.25 ± 0.17	*n*.*s*.

**Table 3:** Thorax transection dimensions. Mean ± SD. n.s. = no significant difference.

	control	NAAI	*p*
Thorax transection depth (cm)	10.1 ± 1.4	9.6 ± 1.9	*n*.*s*.
Thorax transection width (cm)	23.7 ± 1.4	23.9 ± 1.3	*n*.*s*.
Thorax transection area (cm^2^)	187.9 ± 32.0	181.2 ± 43.7	*n*.*s*.

### Aortic arch shape

**Fig 4:**
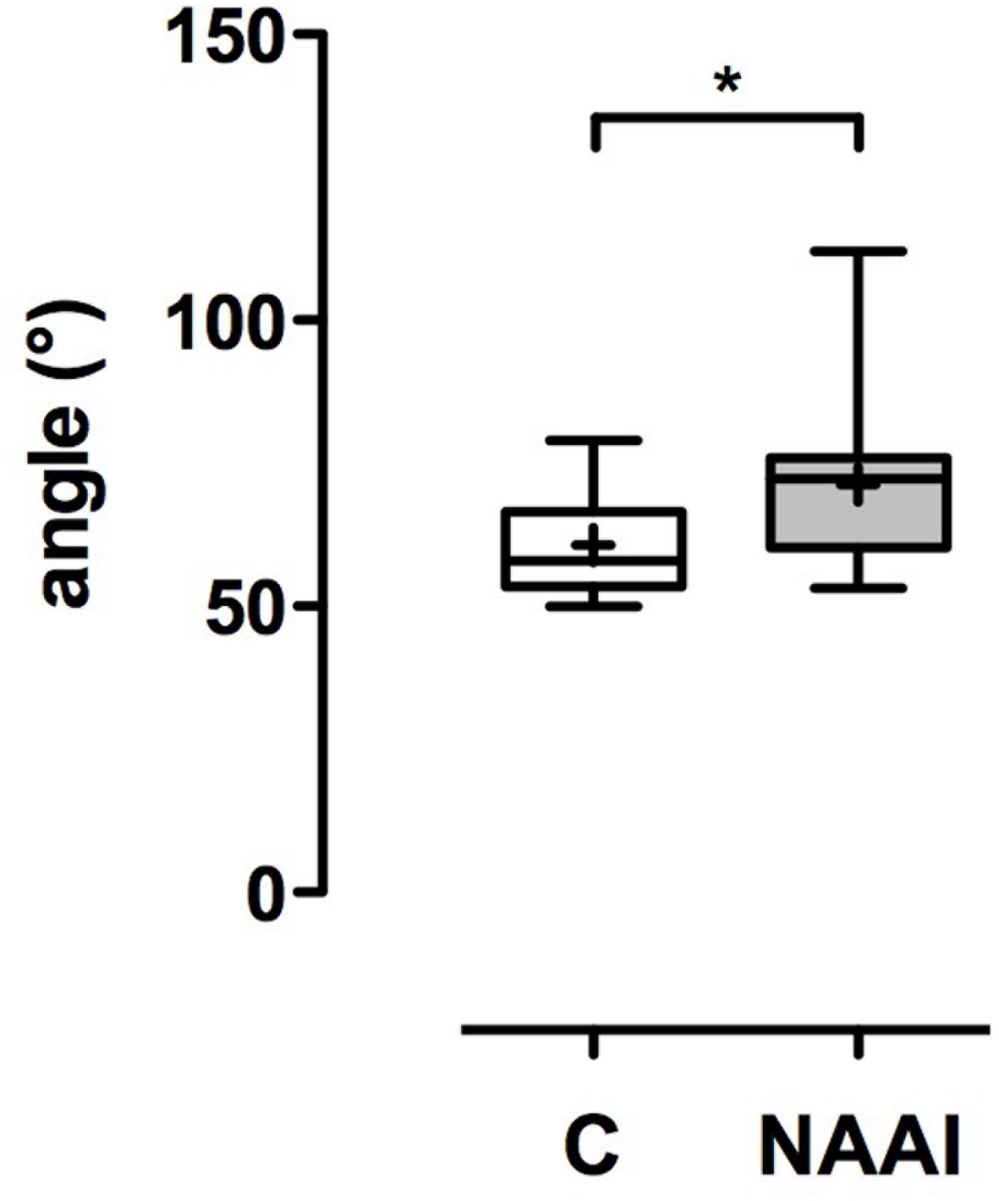
Aortic arch angle (AAA). The AAA is larger in NAAI than in control (p*<0.05).

**Fig 5:**
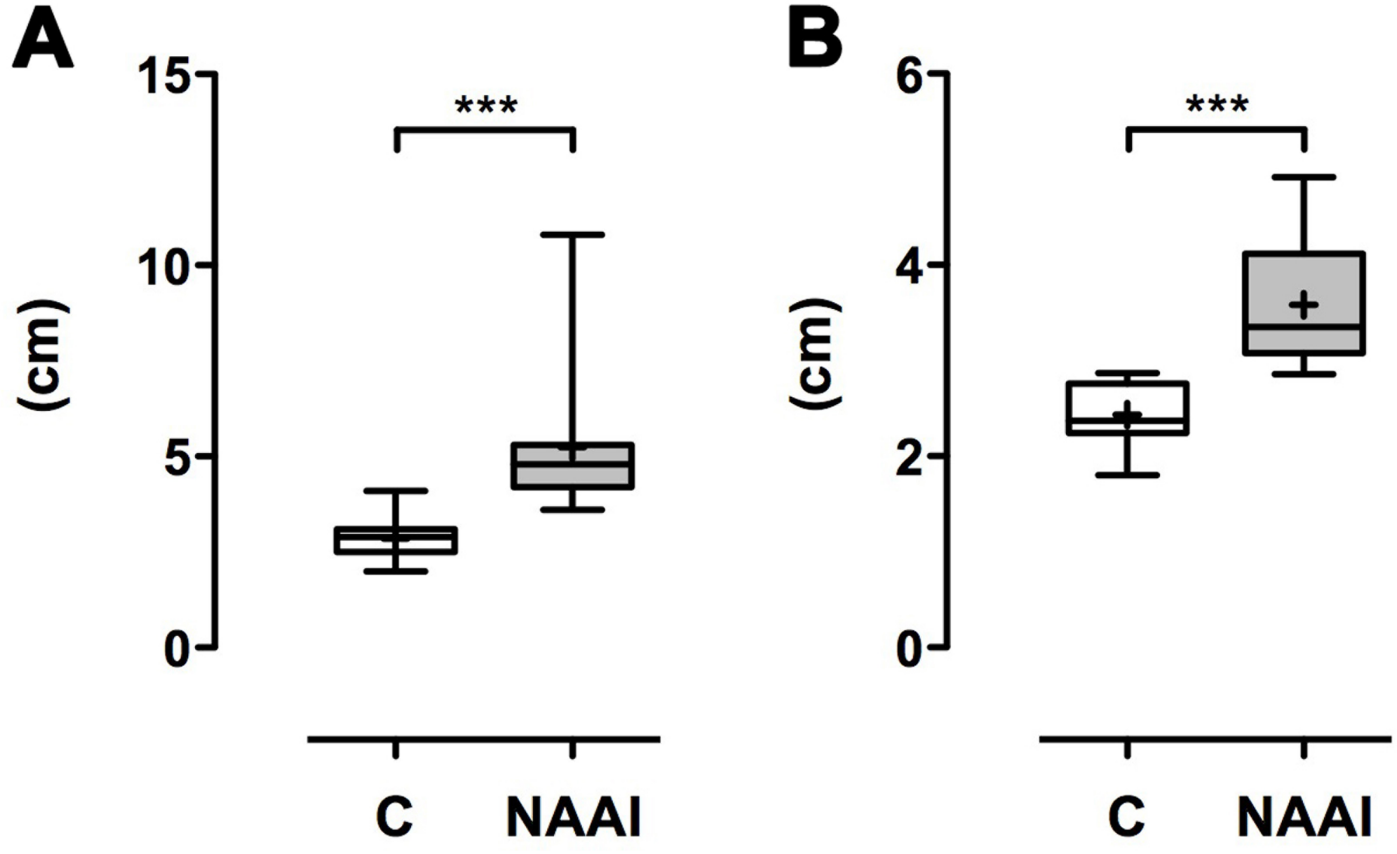
Aortic arch index (AAI) and aortic arch height (AAH). Both the AAI and AAH are significantly larger in patients with NAAI compared to healthy control (p***<0.001).

**Fig 6:**
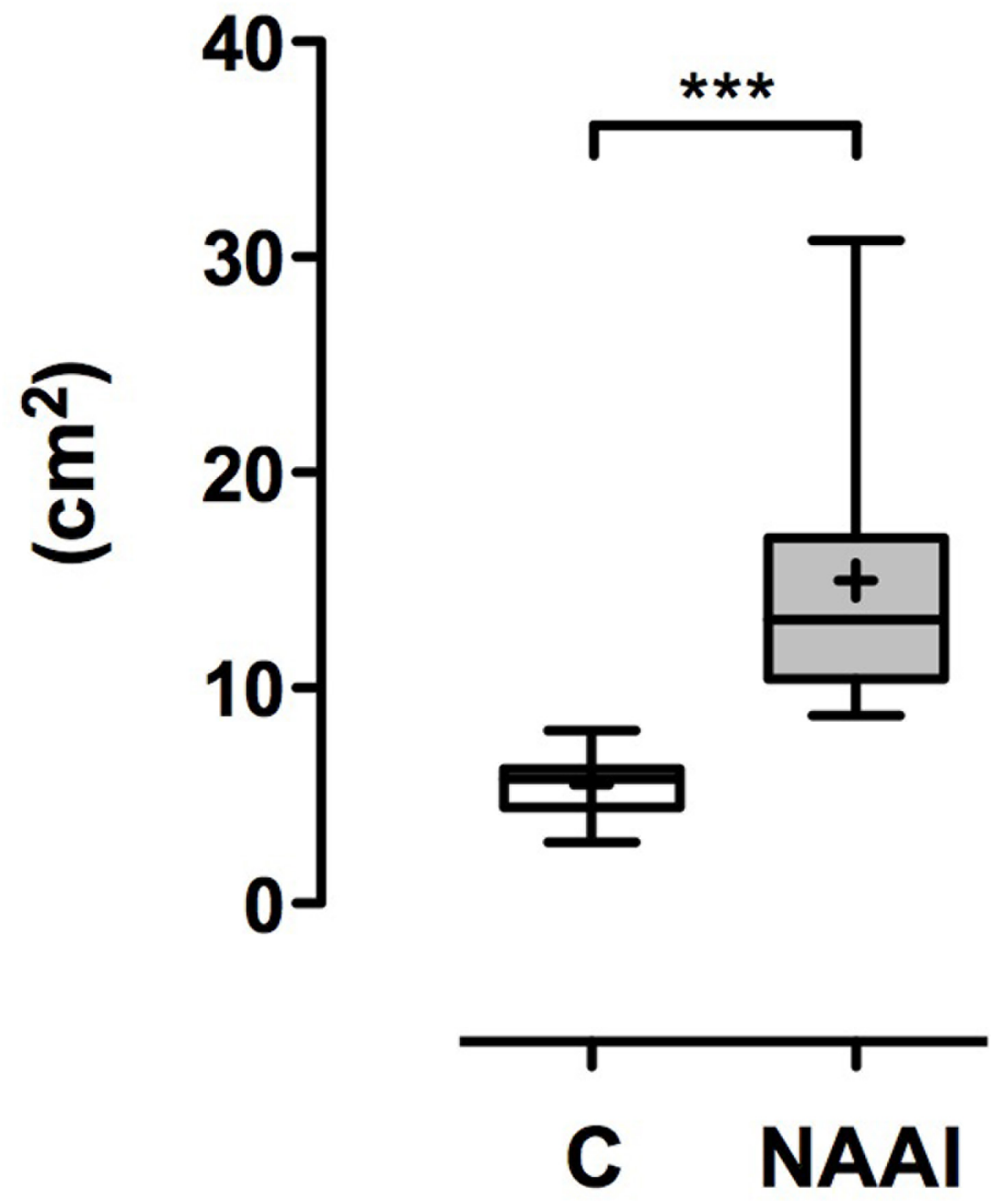
Area under arch (AUA). The area under the aortic arch is significantly larger in patients with NAAI than in individuals without NAAI (p***<0.001).

The AAA in individuals with NAAI (71.3° ± 14.9°) is larger than in healthy control (60.7° ± 8.6°; p*<0.05; Table 4; Fig 4). In patients with NAAI, the distance between ascendent and descendent aorta (AAI) is larger (5.2 cm ± 1.9 cm) than in matched control (2.8 ± 0.5 cm; ***p<0.001; Table 4; Fig 5). Moreover, the aortic arch is higher above tracheal bifurcation in NAAI (3.6 cm ± 0.6 cm) in comparison to matched control (2.4 cm ± 0.3 cm; ***p<0.001; Table 4; Fig 5). Accordingly, the area under the aortic arch (AUA), calculated as half of an eliptic shape from AAH and AAI, is significantly larger in individuals with NAAI (15.0 cm^2^ ± 6.5 cm^2^) when compared to age- and sex-matched controls without NAAI (5.5 cm^2^ ± 1.3 cm^2^; ***p<0.001; Table 4; Fig 6).

**Table 4:** Geometric measures of aortic arch. AAI: distance of A. ascendens and A. descendens. Mean ± SD. p* < 0.05. p*** < 0.001. n.s. = no significant difference.

	control	NAAI	*p*
Aortic arch index (AAI; cm)	2.8 ± 0.5	5.2 ± 1.9	***
Aortic arch angle (AAA; °)	60.7 ± 8.6	71.3 ± 14.9	*
Aortic arch height (AAH, cm)	2.4 ± 0.3	3.6 ± 0.6	***
Area under arch (AUA)	5.5 ± 1.3	15.0 ± 6.5	***

## Discussion

Non-penetrating injuries of the aortic arch are beyond the most fatal in emergency medicine [1]. It was observed, that the risk of NAAI depends on the magnitude of deceleration (*Δv/Δt*), with a higher magnitude of deceleration being more likely to lead to a NAAI [3], and the direction of impact, with a lateral force-vector being more often associated with NAAI [4]. Actually, most sudden decelerations are followed by a sudden re-acceleration; this phenomenon e.g. causes the so-called „whiplash-injury”of the cervical spine in car accidents [8], or the „coup”and „contre-coup”in traumatic intracranial hemorrhage [9]. In the latter, the brain is pushed forwards within the skull at the moment of deceleration (coup), and bounces back against the occiput in an act of re-acceleration (contre-coup), causing two distinct hemorrhages. The heart and great vessels in the mediastinum are believed to behave in a comparable manner within the bony thorax [10]: being pushed forward against the sternum in deceleration, and re-accelerating counter wise in direction to the spine. Based on the theory of superposition, the force on the aortic wall is the sum of different smaller forces, e.g. linear deceleration (*F = m x Δv/Δt*) and the moment of inertia (*I*), which is calculated by multiplying the mass (*m*) of an object by the square of the distance (*r*) to its pivot point (*l = m x r*^*2*^). The mass of the aorta mainly depends on its volume, which can be calculated from the diameter. But in our study, no differences between groups were found regarding the diameter of the aorta, with absolute values being comparable to results previously reported [11]. The second factor contributing by square is the distance to the pivot point (*r*^*2*^). While all four chambers of the heart including the valves, the great vessels (aorta, pulmonary artery, pulmonary veins, vena cava superior et inferior), the lungs, and the esophagus have to be motile, the trachea and the left- and right-main bronchus don’t. Assuming the tracheal bifurcation as a center of rotation, the fulcrums for angular deceleration equal half of AAI and AAH. This observation is backed by statistically significant differences for both, AAI and AAH, between individuals with and without NAAI. Calculated from AAI and AAH, the same is true for the AUA.

Comparably, Wojciechowski et al. made the observation, that the AAA and AAI correlate with the severity of aortic arch injury [7]. As a difference to their measurements taken, we set the top of the triangle for the AAA to the inner wall of the aortic arch to not falsify results by the diameter of the aorta; since the latter showed no difference in between groups, we think both measurements are equally useful. The results of our study have to be discussed in the light of a small cohort size and the single-center design. Moreover, while basic principles of physics definitely apply, simplifications have to be made which are prone to false assumptions (e.g. center of angular deceleration). Further studies, e.g. *ex vivo*, should be undertaken to gain further insights to the development and biomechanics of NAAI. A broad and profound understanding of contributing risk factors is needed for further advances in automotive and road safety [12].

## Conclusion

Development and biomechanics of NAAI aren’t yet fully understood. Magnitude of deceleration and direction of impact were the only factors known to influence the risk for NAAI so far. We now propose the height and width of aortic arch as 3^rd^ and 4^th^ factor in the development of traumatic NAAI, assuming an angular deceleration (and re-acceleration) component. As traffic accidents are the most common source of a deceleration/acceleration trauma, further advances in automotive and road safety need a detailed understanding of the biomechanics underlying NAAI. This requires further experimental studies to shed light on the development of NAAI.

## Abbreviations

A.: Aorta
AAA: Aortic arch angle
AAH: Aortic arch height
AAI: Aortic arch index (= width)
AUA: Area under aortic arch
CT: Computed tomography
FoV: Field of view
ISS: Injury severity score
NAAI: non-penetrating aortic arch injury
